# Prevalence and predictors of depression in tuberculosis patients in india: a systematic review and meta-analysis

**DOI:** 10.1007/s44192-025-00248-9

**Published:** 2025-07-12

**Authors:** Janmejaya Samal, Ranjit Kumar Dehury, M. Benson Thomas, Hari Singh

**Affiliations:** 1 School of Public Health, SRM Institute of Science and Technology, Kattankulathur, Tamil Nadu India; 2 School of Management Studies, University of Hyderabad, Hyderabad, Telangana India

**Keywords:** Common mental health disorders, Drug-Resistant TB, Drug-Sensitive TB, Predictors, Pulmonary TB

## Abstract

**Introduction:**

TB and common mental disorders pose significant global health challenges that considerably impact human health. The combination of depression with TB can lead to a poor quality of life, low medication adherence, progression to drug-resistant tuberculosis, and ultimately, mortality.

**Objectives:**

This study aimed to estimate the pooled prevalence of depression in TB patients and identify the predictors of depression in this population in India.

**Methods:**

The preferred Reporting Items for Systematic Reviews and Meta-Analyses (PRISMA) guidelines were followed for reporting this systematic review and meta-analysis. Data were extracted from October to December 2024 using the PUBMED, Scopus, EMBASE, and DOAJ databases. A total of 25 articles were selected, and the included articles underwent quality assessment using the Joanna Briggs Institute Critical Appraisal checklist. The pooled prevalence of depression in TB patients was estimated at a 95% confidence interval using a random effects model, assuming potential heterogeneity. STATA 18 (Stata Corp LLC, College Station, TX, USA) was used for analysis.

**Results:**

The total sample across 25 studies included 12,033 (Mean(SD) = 481(1377), Median = 169, IQR = 106–302). The pooled prevalence of depression in TB patients in India was estimated at 37% (95% CI: 26- 49%). A subgroup analysis based on the types of TB cases indicated that the prevalence of depression in different kinds of TB cases did not vary substantially, with 39% (95% CI: 26- 54%) in both Drug-Resistant (DR) and Drug-Sensitive (DS) Tuberculosis (TB) cases, followed by DR-TB cases [36% (95% CI: 09-68%)] and DS-TB cases [32% (95% CI: 14- 53%)]. Of the nine assessment tools used to assess depression, the pooled prevalence utilising the Patient Health Questionnaire (PHQ)-9 tool was highest [43% (95% CI: 31-56%)]. There was considerable heterogeneity (I^2^ = 99.10%) observed in the random-effects model. Factors associated with depression in TB patients included gender, demographics, education, occupation, marital and relationship issues, religion, socio-economic status, habitat, disease-related factors, treatment-related factors, and social and Behavioural factors.

**Conclusion:**

The study found that over one-third of TB patients experienced depression. The coexistence of depression and TB constitutes a significant public health issue that needs addressing at both the community and health facility levels.

## Introduction

Tuberculosis is the leading cause of death from a single infectious disease in the world. It is caused by the bacillus Mycobacterium tuberculosis, which is transmitted when infected individuals aerosolise bacteria into the atmosphere, such as through coughing [1]. The disease is preventable and usually curable; however, without treatment, the death rate can be as high as nearly 50%. With the current WHO-recommended treatment regimen lasting 4–6 months, the cure rate reaches almost 85%. According to the Global TB burden report-2024, 87% of the TB burden originates from the top 30 high-burden countries, with 56% coming from the five highest-burden countries, including India, which alone accounts for 26% of the total global burden [1]. It has caused almost twice as many deaths as HIV/AIDS. As estimated, more than 10 million people continue to fall ill every year, and numbers have been increasing since 2021 [1]. Furthermore, the TB India report states that in 2023, the incidence and mortality of TB in India were 199 and 23, respectively, compared to targets of 77 and 6 [2]. 

A large proportion of the global population that TB latently infects provides a reservoir for the upcoming active TB cases coming from the underdeveloped world that is linked with poverty, absence of healthy living conditions and inadequate medical care [3, 4]. The association of common mental disorders such as depression, anxiety, and somatoform disorders is another set of added problems to existing poverty [5]. TB and common mental disorders are global health challenges that have a considerable impact on human health. Both these conditions share common determinants such as drug addiction, poverty and homelessness. Despite this, the relationship has received less attention around the globe. Furthermore, higher morbidity and mortality are seen among patients with baseline psychiatric illnesses as they default on the treatment [6]. Reports suggest that there is a high prevalence of psychiatric disorders in TB patients. However, screening for the same among primary care physicians and specialists is not a usual practice [7]. 

Of all the common mental disorders, depression among TB patients seems to be highly prevalent, as per various reports [8–11]. Depression is a significant mental health condition expected to become the most debilitating and common health issue by 2030; notably, tuberculosis is a frequent comorbidity associated with depression. Consequently, considerable attention has been given to the association between these two illnesses. Clinical data show a bidirectional relationship between tuberculosis and depression, characterised by a notable overlap in symptoms. Infection or reactivation of tuberculosis may trigger depression, likely due to the body’s inflammatory response and/or disruption of the hypothalamic–pituitary–adrenal axis. Additionally, hypotheses suggest a correlation between depression and tuberculosis, highlighting the role of the immune-inflammatory response and lipid metabolism as potential pathways [12]. 

Research suggests that emotional distress, depression, and anxiety are important dimensions of TB and are closely linked to the severity of symptoms, the number of symptoms reported, higher rates of health care utilisation, poor treatment adherence, longer treatment duration, decreased disease control, and mortality [13, 14]. It was also found that long-term and complex treatment associated with TB can lead to mental, emotional and psychiatric disorders [15, 16]. The combination of depression with TB can lead to poor quality of life, poor medication adherence, progression to multidrug-resistant TB, and finally ending up with mortality [17, 18]. 

Several observational studies across the globe have estimated the prevalence of depression among TB patients, which ranged from 14.3 to 49.3% [7, 15, 16, 19–21]. Similarly, a few systematic reviews have also been carried out on this subject. A systematic review considering the global data has estimated the prevalence of depression among TB patients at 11% (95% CI: 11-12%) [22], and another systematic review has estimated the prevalence of depression among South-Asian MDR-TB cases at 54% (95% CI: 42-65%) [23]. However, this systematic review and meta-analysis was primarily aimed at assessing the predictors and estimating the pooled prevalence of depression among Indian patients.

### Objective

The main objectives of this systematic review and meta-analysis were to estimate the pooled prevalence of depression and assess the predictors of the same in TB patients in India.

## Materials and methods

Preferred Reporting Items for Systematic Reviews and Meta-Analyses (PRISMA) guidelines were followed to report this systematic review and meta-analysis. ^[24]^ This section describes the data sources, search strategies and the inclusion and exclusion criteria.

### Information sources and search strategy

The PUBMED, Scopus, DOAJ and EMBASE databases were used to identify the studies. The articles were screened during November and December 2024 (up to 31st December). Table 1 describes the search details used for this systematic review and meta-analysis.

**Table 1:** Search details for the extraction of articles for different databases

Databases	Results	Search details
Scopus	76	( TITLE-ABS-KEY ( ”tuberculosis” OR ”TB” ) AND TITLE-ABS-KEY ( ”depression” OR ”depressive disorder” OR ”major depressive disorder” OR ”mental health” OR ”psychological distress” ) AND TITLE-ABS-KEY ( ”prevalence” OR ”epidemiology” OR ”frequency” OR ”occurrence” ) AND TITLE-ABS-KEY ( ”India” OR ”Indian” ) )
EMBASE	41	Sources Embase, MEDLINE, Preprints Query(‘tuberculosis’:ab, ti OR ‘tb’:ab, ti) AND (‘depression’:ab, ti OR ‘depressive disorder’:ab, ti OR ‘major depressive disorder’:ab, ti OR ‘mental health’:ab, ti OR ‘psychological distress’:ab, ti) AND (‘prevalence’:ab, ti OR ‘epidemiology’:ab, ti OR ‘frequency’:ab, ti OR ‘occurrence’:ab, ti) AND (‘india’:ab, ti OR ‘indian’:ab, ti) Mapped terms n/a
PubMed	24	(“Prevalence”[MeSH Terms] OR “Epidemiology”[MeSH Terms] OR “Prevalence”[Title/Abstract]) AND (“Tuberculosis”[MeSH Terms] OR “Tubercuolosis”[Title/Abstract] OR “TB”[Title/Abstract]) AND (“Depressive Disorder”[MeSH Terms] OR “Depression”[MeSH Terms] OR “Mental Health”[MeSH Terms] OR “Depression”[Title/Abstract]) AND (“india”[MeSH Terms] OR “india”[All Fields] OR “india s”[All Fields] OR “indias”[All Fields])
DOAJ	58	(Depression) AND (Tuberculosis) AND (India) [All fields]

### Study selection

Zotero 7.0.7 (corporation for digital scholarship) was used for deduplication [25]. The articles were then screened with the titles for the first round of screening. The remaining articles were then independently reviewed by the first two authors, who involved reading abstracts and reviewing the full text of the articles. The final inclusion of the articles was based on a mutual consensus of all the authors.

### Eligibility criteria

The eligibility criteria of the studies are decided based on the PICOS format and are described in Table 2.

**Table 2:** PICOS framework for the eligibility of studies

Criteria	Description of criteria
Population	TB cases in India (All types of TB cases; DS, DR and Both DS & DR-TB cases)
Intervention/Exposure	Not applicable
Comparison	Not applicable
Outcome	Prevalence of Depression in TB patients
Study Designs	Observational studies

### Inclusion and exclusion criteria

The following criteria were decided for the inclusion and exclusion of the studies, are described in Table 3.

**Table 3:** Inclusion and exclusion criteria of studies

Parameters	Inclusion criteria	Exclusion criteria
Language	English language	Other than English language
Country	India	Other than India
Study design	Primary research	Reviews/commentaries/opinions/correspondences
Publication status	Published articles	Articles in press or pre-published articles
Study focus	Prevalence of Depression in TB patients	Other mental health conditions

### Quality assessment of included studies

Once the screening was over, the full-text articles were evaluated individually using the Joanna Briggs Institute Critical Appraisal Tools Checklist for cross-sectional studies ^[24]^ to ascertain the superiority of these studies, as depicted in Table 4. The checklist has eight items:

1. Were the criteria for inclusion in the sample clearly defined?
2. Were the study subjects and the setting described in detail?
3. Was the exposure measured in a valid and reliable way?
4. Were objective, standard criteria used for measurement of the condition?
5. Were confounding factors identified?
6. Were strategies to deal with confounding factors stated?
7. Were the outcomes measured in a valid and reliable way?
8. Was appropriate statistical analysis used?

Answers such as yes, no, unclear, or not applicable were assigned to each item and were colour-coded as depicted in Table 4. The score was calculated, and the quality of these studies was categorised based on the scores: < 50%-Poor quality, 50-75%-Moderate quality, > 75%-Good quality.

**Table 4:**
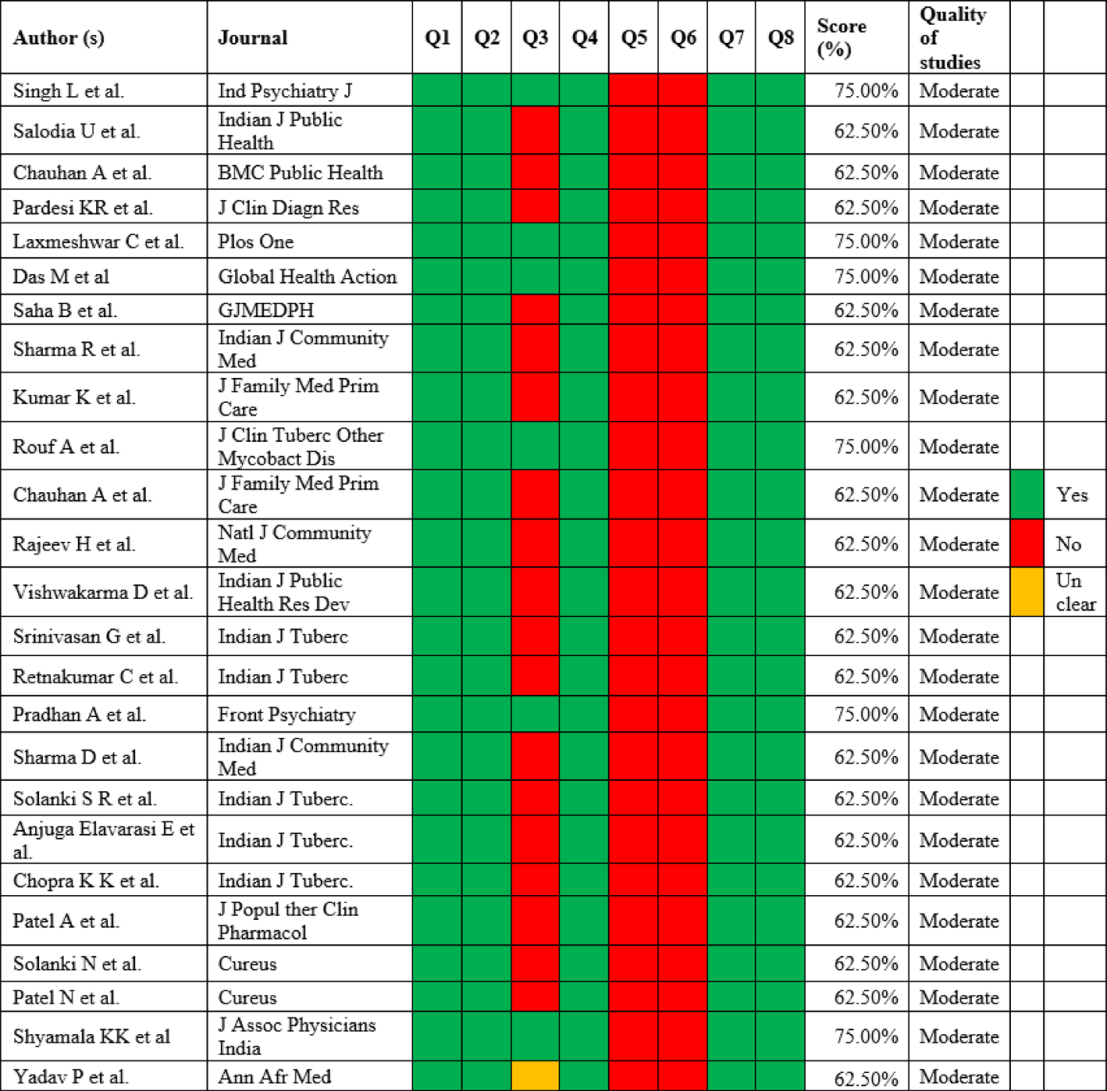
The Joanna Briggs Institute critical appraisal tools, checklist for cross-sectional studies

### Data extraction

The basic details, such as the author’s name, journal name, and the study setting/Indian state where the study was conducted, were documented. Further, the sample size, study participants’ age, study design, assessment scale used, prevalence of depression, and predictors were also noted.

### Statistical analysis

Using a random effect model that assumed potential heterogeneity, the pooled prevalence of depression in TB patients in India was calculated at a 95% confidence interval. The bias and variability within the included studies were graphically represented using a funnel diagram, forest plot and Galbraith plot. In addition, non-parametric trim-and-fill analysis, regression-based Egger’s test for small study effect, and leave-one-out sensitivity analysis were employed for further analysis of publication bias and heterogeneity. The threshold for statistical significance was set at *p* < 0.05. STATA18 (Stata Corp LLC, College Station, TX, USA) was used for analysis.

## Results

### Study selection

**Fig. 1:**
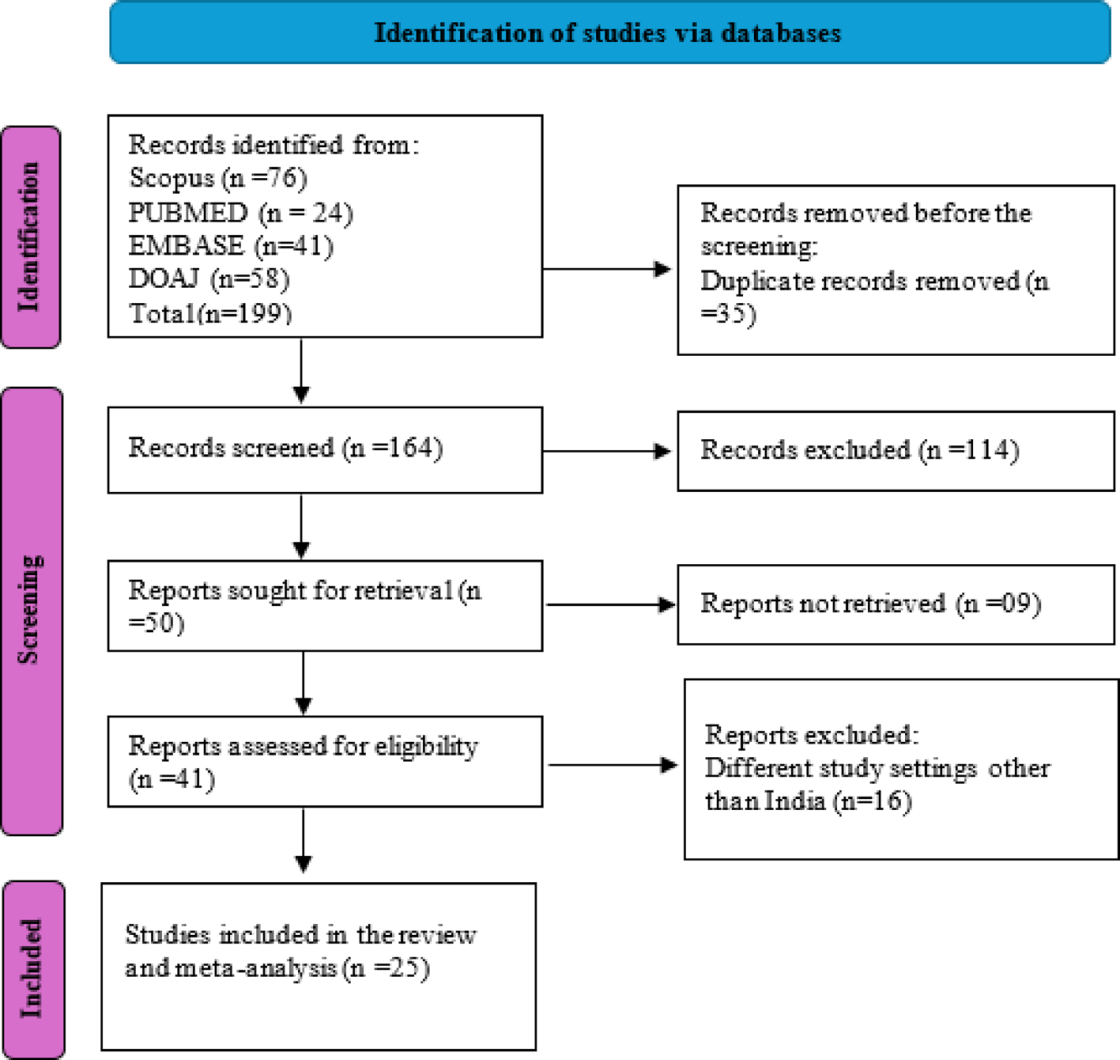
PRISMA flow-chart

Of the 199 identified articles, 35 were removed as duplicates, and 164 were screened, of which 114 were excluded. Fifty articles were sought for retrieval; nine studies could not be retrieved, and 16 studies were excluded for not meeting the eligibility criteria, leading to the inclusion of 25 articles for the review. Figure 1 shows the PRISMA flow chart depicting the stages of identification, screening, and final inclusion of the articles.

### Study characteristics

All the included studies (*n* = 25) are observational studies, with the majority of studies (*n* = 21) being cross-sectional, and two studies are of retrospective design, while one each is of prospective and hospital-based longitudinal design in nature. The studies were distributed across different Indian states and union territories, with 14 unique states and union territories. The highest representation came from Gujarat (*n* = 5), Uttar Pradesh (*n* = 4) and Karnataka (*n* = 3), followed by Delhi and Maharashtra with two studies each, and the rest of the states had one study each. Geographically, the studies represented several zones of India, North India (*n* = 4), South India (*n* = 4), East India (*n* = 3), West India (*n* = 2) and Central India (*n* = 1). The included studies employed different assessment tools, in which the Patient Health Questionnaire (PHQ-9) was used in the maximum (*n* = 13) instances, followed by other tools as listed in Table 5. The reviewed articles were published between 2014 and 2024, with a substantial increase in publication in recent years, in which nine relevant articles were published in 2024, followed by five in 2022. All the studies used the adult population aged 18 years and above (*n* = 23), and one study used the population aged 15 years and above. Different types of TB cases were used as study participants, in which all types of TB cases (both DS and DR-TB cases) were used 16 times, DS-TB cases (*n* = 5) and DR-TB cases (*n* = 4).

### Result of individual studies

**Table 5:** Results of individual studies included in the review

Author (s)	Journal	YOP	Study setting	Study duration	Sample size	Study participants and age	Study design	Prevalence of Depression	Assessment tool
Singh L et al. [27]	Ind Psychiatry J	2015	Uttar Pradesh	July 2014-June 2015	100	Both DS and DR-TB cases, 18 years and above	Cross-sectional	44% (*n* = 44)	BDI
Salodia U et al. [28]	Indian J Public Health	2019	Delhi	January to March 2018	106	DS-TB, 18 years and above	Cross-sectional	23.6% (n = 25)	PHQ-9
Chauhan A et al. [29]	BMC Public Health	2024	Telengana and Odisha	October 2022 to March 2023	323	Both DS and DR-TB cases, 18 years and above	Cross-sectional	33% (*n* = 107)	MAQ-PC
Pardesi KR et al. [30]	J Clin Diagn Res	2020	Andhra Pradesh	October 2013 to September 2015	120	Both DS and DR-TB cases, 18 years and above	Cross-sectional	43.33% (*n* = 52)	MINI Scale
Laxmeshwar C et al. [31]	Plos One	2022	Maharashtra	Not reported	119	DR-TB cases, 18 years and above	Retrospective study	41.1% (*n* = 49)	PHQ-9
Das M et al. [32]	Global Health Action	2014	Maharsshtra	August 2012 through to March 2014	45	DR-TB cases, > 15 years of age	Retrospective study	16% (*n* = 7)	PHQ-9
Saha B et al. [33]	GJMEDPH	2023	Tripura	January 2020 to June 2021	120	Both DS and DR-TB cases, 18 years and above	Cross-sectional study	21.7% (*n* = 26)	MINI Scale
Sharma R et al. [34]	Indian J Community Med	2022	Gujarat	Not reported	185	DR-TB cases	Cross-sectional study	16.2% (*n* = 30)	HAM-D Scale
Kumar K et al. [35]	J Family Med Prim Care	2016	Uttar Pradesh	February 2015 to November 2015	100	Both DS and DR-TB cases, 20 years and above	Cross-sectional study	35% (*N* = 35)	BDI-II
Rouf A et al. [36]	J Clin Tuberc Other Mycobact Dis	2021	Jammu and Kashmir	1st April 2017 to 30th September2018	202	DS-TB, 18 years and above	Prospective study	50.5% (*n* = 102)	PHQ-9
Chauhan A et al. [37]	J Family Med Prim Care	2024	Gujarat	October 2021 to July 2023	7056	Both DS and DR-TB cases, 18 years and above	Cross-sectional study	5% (*n* = 379)	PHQ-4
Rajeev H et al. [38]	Natl J Community Med	2022	Karnataka	January 2020 to December 2021	302	Both DS and DR-TB cases, 18 years and above	Cross-sectional study	45.7% (138)	Zung Self-Rating Depression Scale
Vishwakarma D et al. [39]	Indian J Public Health Res Dev	2020	Madhya Pradesh	March to May 2018	103	DS-TB, 18 years and above	Cross-sectional	64% (*n* = 66)	PHQ-9
Srinivasan G et al.[40]	Indian J Tuberc	2021	Uttar Pradesh	February to December 2019	263	DR-TB cases, 18 years and above	Cross-sectional	73% (*n* = 192)	PHQ-9
Retnakumar C et al. [41]	Indian J Tuberc	2022	Kerala	December 2019 to March 2020	485	Both DS and DR-TB cases, 18 years and above	Cross-sectional	16.1% (*n* = 78)	PHQ-9
Pradhan A et al. [42]	Front Psychiatry	2022	Sikkim	January 2019 and July 2020	71	Both DS and DR-TB cases, 18 years and above	Cross-sectional	56.3% (*n* = 40)	PHQ-9
Sharma D et al. [43]	Indian J Community Med	2024	Chandigarh	December 2022 to February 2023	305	DS-TB, 18 years and above	Cross-sectional	7.2% (*n* = 22)	PHQ-9
Solanki S R et al. [44]	Indian J Tuberc	2024	Gujarat	Started late 2019 and completed in 2021	600	Both DS and DR-TB cases, 18 years and above	Cross-sectional	41.2% (*n* = 247)	HADS
Anjuga Elavarasi E et al. [45]	Indian J Tuberc	2024	Karnataka	NA	169	Both DS and DR-TB cases, 18 years and above	Cross-sectional	57.8% (*n* = 98)	PHQ-9
Chopra K K et al. [46]	Indian J Tuberc	2024	Delhi	NA	47	Both DS and DR-TB cases, 22 years and above [Homeless cases]	Cross-sectional	95.7% (*n* = 45)	PHQ-9 and CESD-R-10
Patel A et al. [47]	J Popul ther Clin Pharmacol	2024	Gujarat	July to November 2022	425	DS-TB, 18 years and above	Cross-sectional	23.29% (*n* = 99)	PHQ-9
Solanki N et al. [48]	Cureus	2023	Madhya Pradesh	September 2020 to January 2021	106	Both DS and DR-TB cases, 18 years and above	Cross-sectional	55.7% (*n* = 59)	PHQ-9
Patel N et al. [49]	Cureus	2024	Gujarat	23 March 2023 and 23 March 2024	272	Both DS and DR-TB cases, 18 years and above	Cross-sectional	36.0% (*n* = 98)	PHQ-9
Shyamala KK et al. [50]	J Assoc Physicians India	2018	Karnataka	June 2016 to October 2016	262	Both DS and DR-TB cases, 18 years and above	Cross-sectional	40.83% (*n* = 107)	PHQ-9
Yadav P et al.[7]	Ann Afr Med	2024	Uttar Pradesh	June 2021 and December 2022	147	Both DS and DR-TB cases, 18 years and above	Hospital‐based longitudinal study	14.3% (*n* = 21)	Brief Psychiatric Rating Scale

#CESD-R-10- Centre for Epidemiologic Studies Depression scale-Revised-10, BDI-The Beck Depression Inventory, HADS-Hospital Anxiety and Depression Scale, HAM (Hamilton Depression Rating Scale)-D Scale, MAQ-PC- Multimorbidity Assessment Questionnaire - Primary Care, MINI- Mini International Neuropsychiatric Interview scale, PHQ-Patient Health Questionnaire.

### Result synthesis

The total sample across 25 studies included 12,033 (mean(SD) = 481(1377), Median = 169, IQR = 106–302). The highest sample used was 7056 in a study in Gujarat, and the lowest sample used was 45 in a study in Maharashtra. The studies reported several factors associated with the prevalence of depression in TB patients in India, which are classified into 12 different categories and are presented in Table 6.

**Fig. 2:**
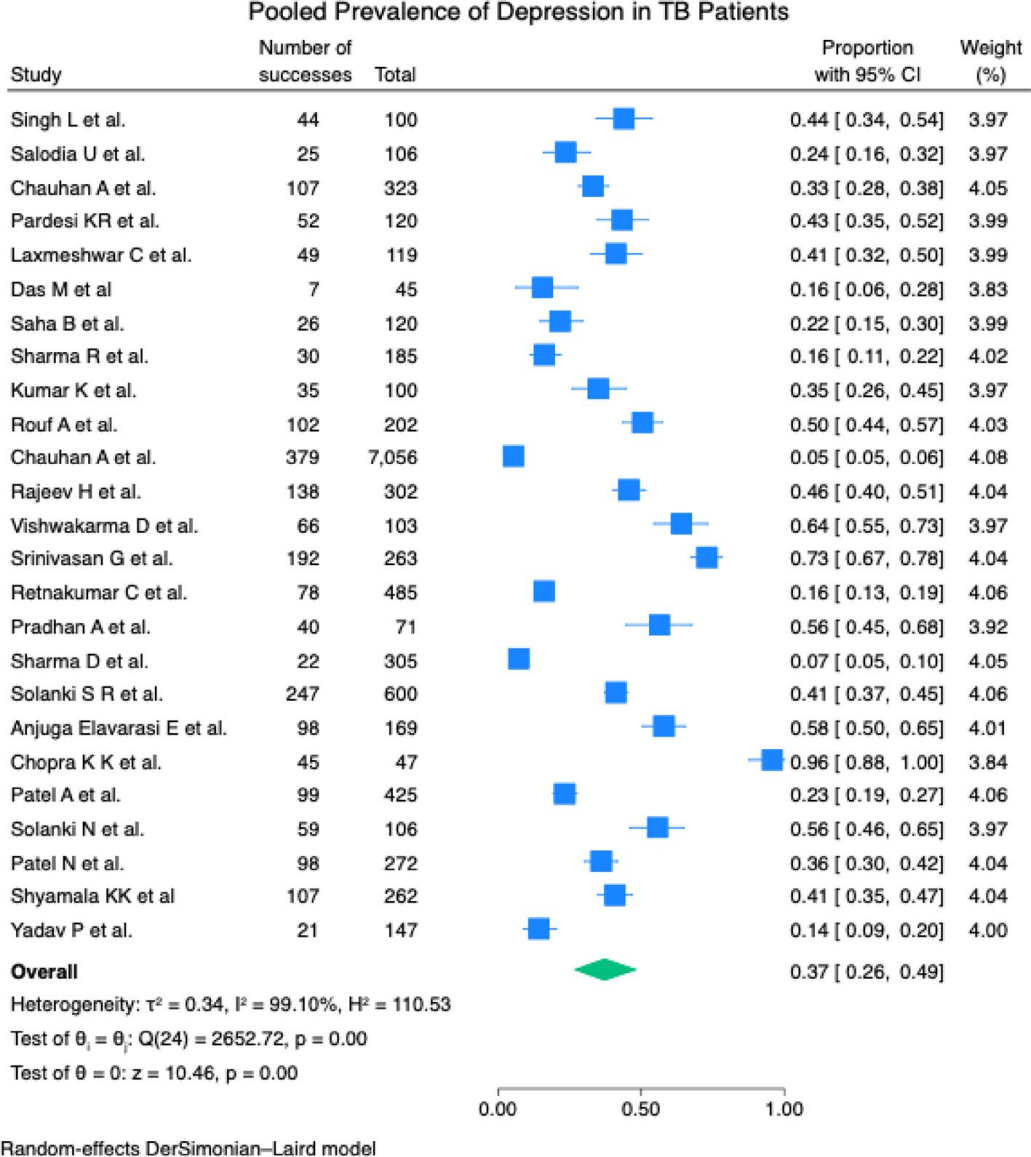
Forest plot showing the pooled prevalence of depression in TB patients

**Fig. 3:**
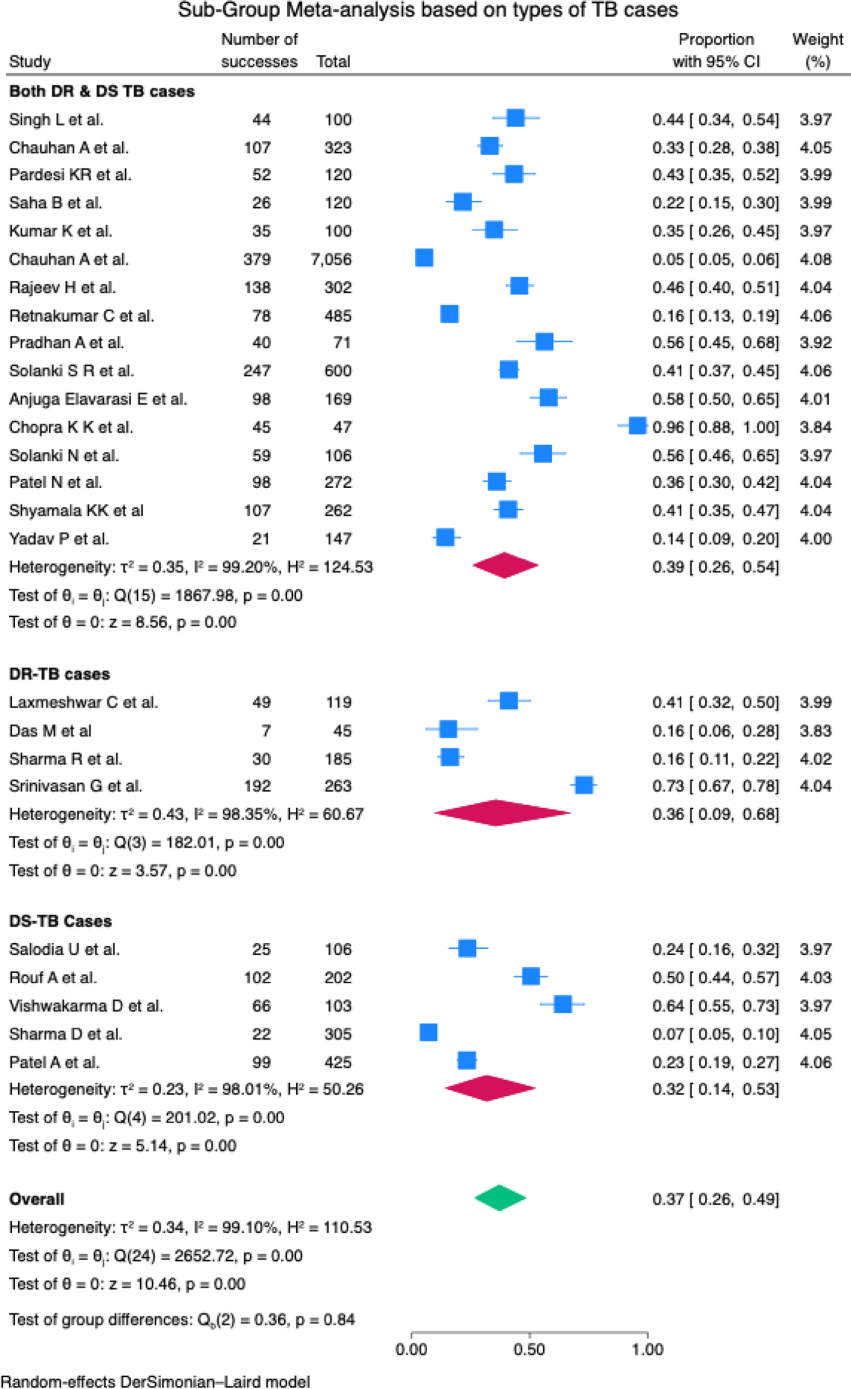
Forest plot showing the subgroup analysis of different types of TB cases

**Fig. 4:**
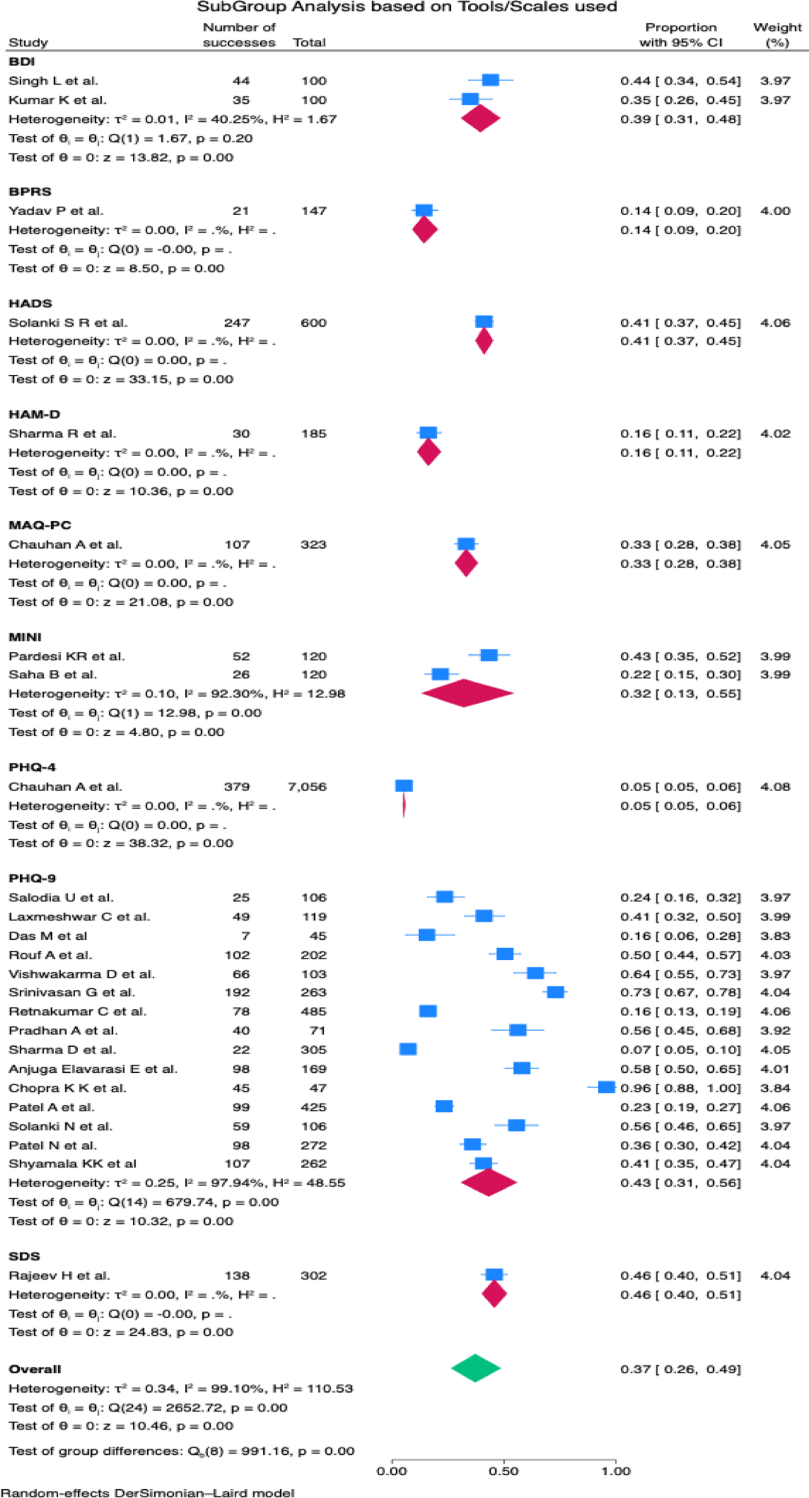
Forest plot showing the subgroup analysis based on the depression tools used

The pooled prevalence of depression in TB patients in India was estimated at 37% (95% CI: 26- 49%). There was considerable heterogeneity (I^2^ = 99.10%), which justified the use of a random effect model. The studies exhibited varying degrees of effect, in which the effect size ranged from 0.05 by Chauhan A et al. to 0.96 by Chopra KK et al. [Fig. 2]. The total effect size (0.37) by the random effect model is significant (z = 10.46, *p* = 0.00), falling between the lower and higher estimates of individual studies. A subgroup analysis based on the types of TB cases indicated prevalence of 39% (CI: 26-54%) in both DR and DS TB cases, followed DR- TB cases [36% (CI: 09- 68%)], DS- TB cases [32% (CI: 14- 53%)] [Fig. 3]. Of the nine assessment tools used to assess depression, the pooled prevalence utilising the PHQ-9 tool was highest [43% (CI: 31-56%)][Fig. 4].

**Fig. 5:**
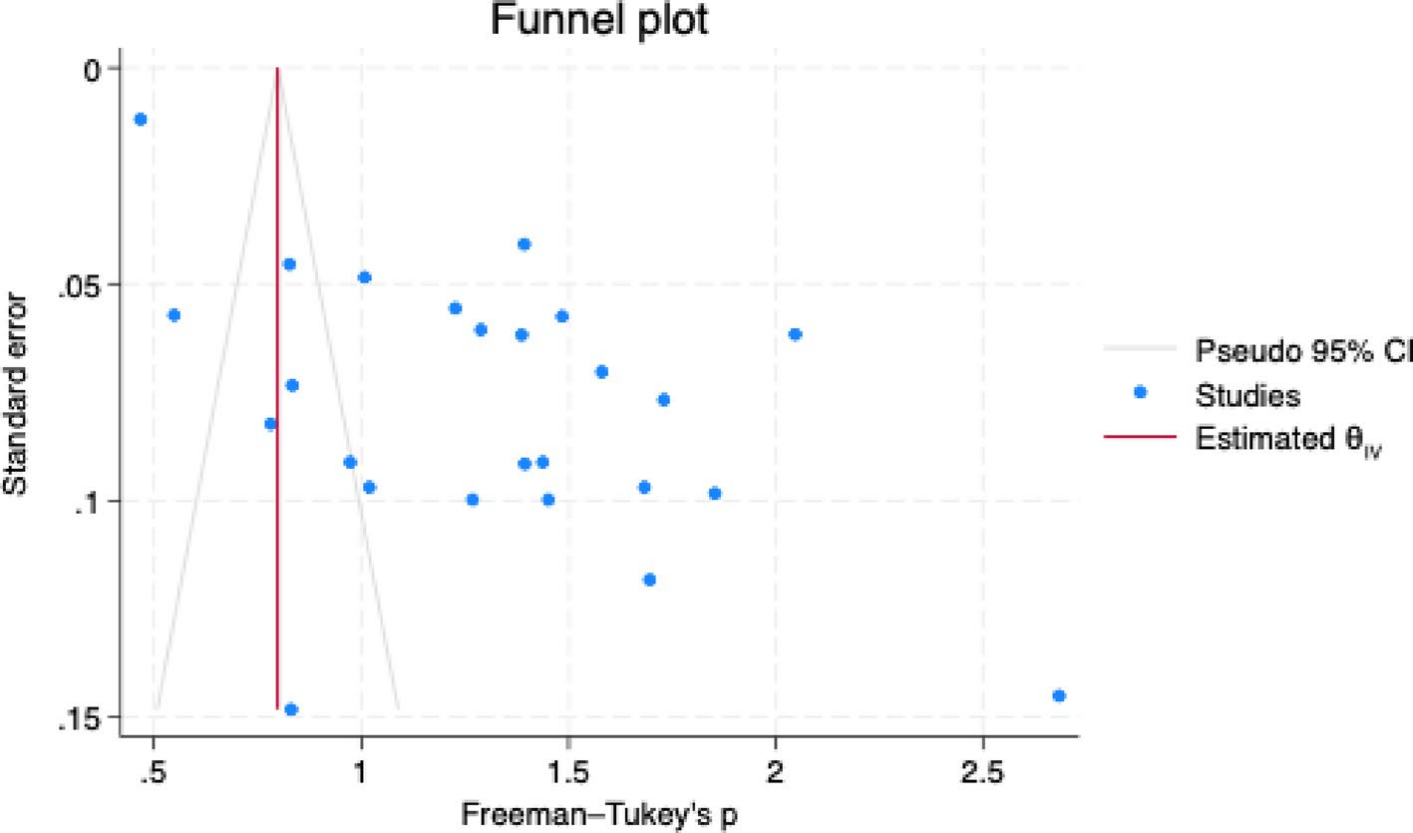
Funnel diagram showing the publication bias

The regression-based Egger’s test for small study effect was employed to assess the publication bias, which is significant at *p* = 0.0182 (z = 2.36) and is also evident in the funnel plot [Fig. 5]. The appearance of the funnel plot suggests a clear asymmetry where a maximum number of studies are on the right side of the pooled estimate. To further assess the publication bias, the trim-and-fill method was employed. The method found that no additional studies were imputed [(Observed: theta = 1.313, 95% CI: 1.083–1.543), (Observed + Imputed: theta = 1.313, 95% CI: 1.083–1.543)], which indicates that the publication bias is unlikely to have a significant impact on the pooled estimate. In this analysis, “theta” represents the overall Freeman-Turkey’s p. The discrepancy in findings of Egger’s test and the trim-and-fill method suggests that the publication bias detected using Egger’s test may be a false positive one or due to the small study effects rather than selective publication.

**Fig. 6:**
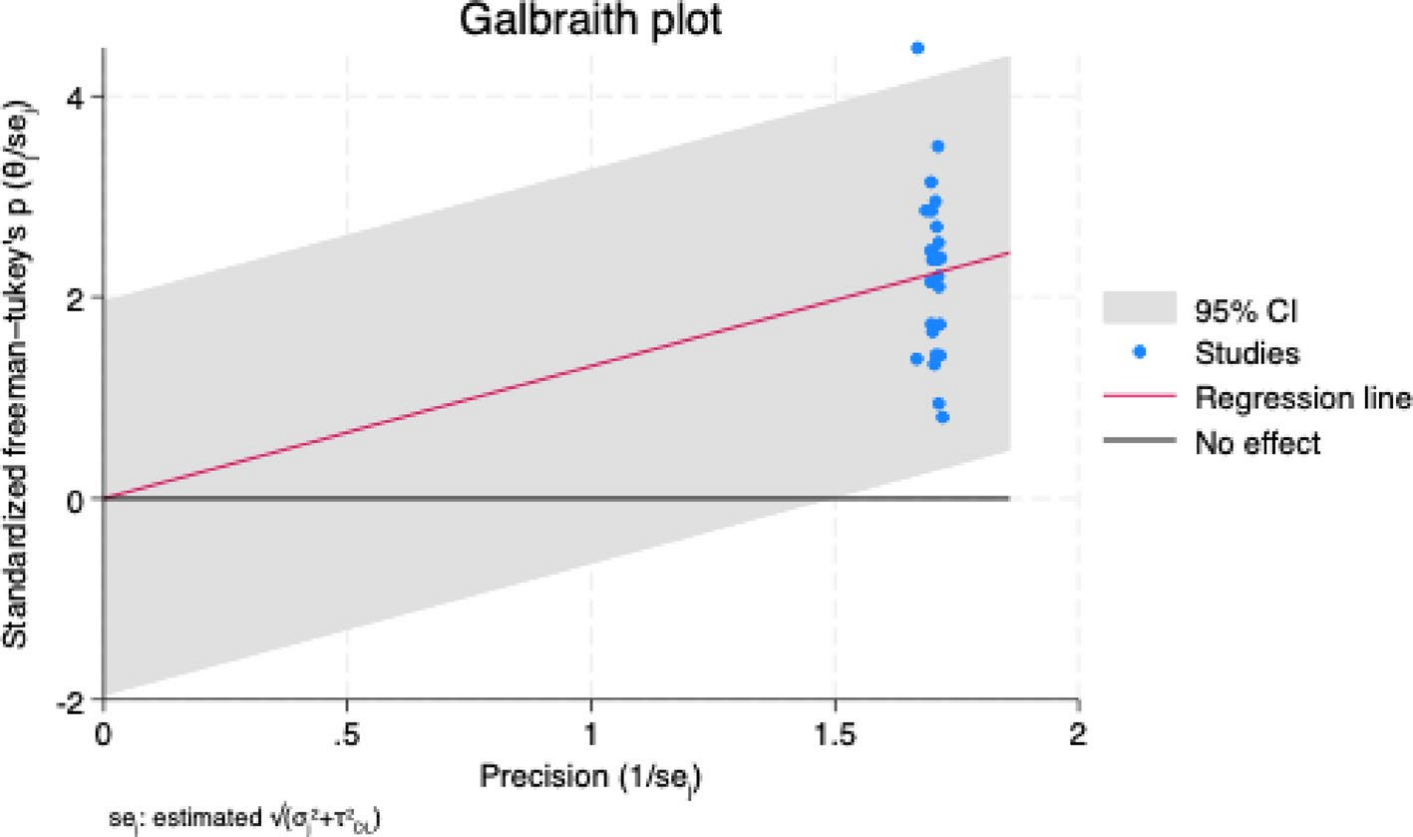
Galbraith plot showing heterogeneity

**Fig. 7:**
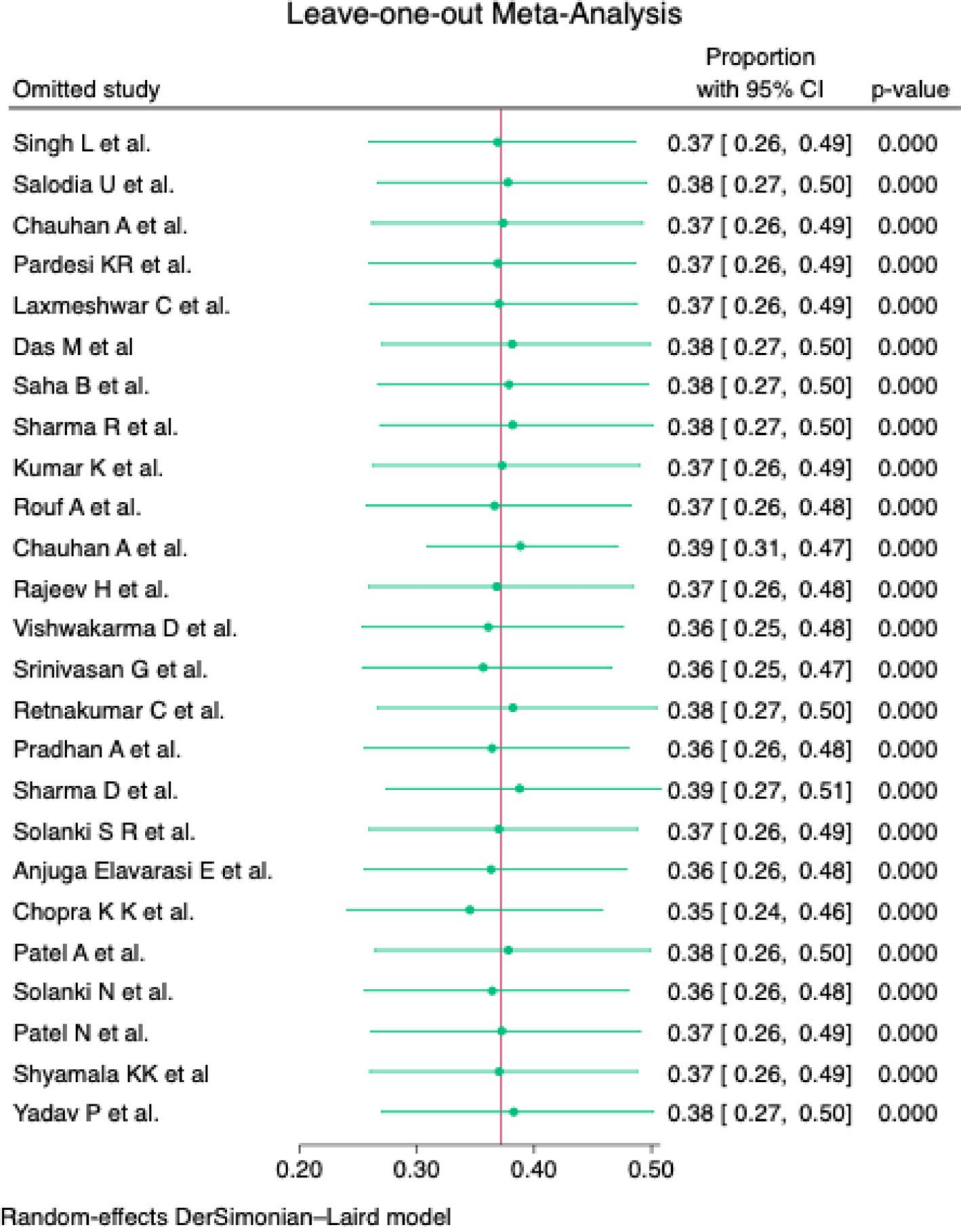
Sensitivity analysis using leave-one-out meta-analysis

In the Galbraith plot, despite the high I^2^ value, most studies conform to the pooled estimate with only one study deviating [Fig. 6]. To further substantiate the same, the sensitivity leave-one-out meta-analysis [Fig. 7] was carried out. The analysis shows that the estimates range between 0.35 and 0.38, and this narrow range indicates that no single study unduly influenced the overall meta-analysis results.

Furthermore, using two study-level covariates, a meta-regression analysis was conducted using total sample size and number of events, in which it was found that the total sample size is significantly associated with the effect size (coefficient=-0.000136, *p* = 0.022), with R-Squared of 52.57%, suggesting 52.57% between-study variance. This suggests that smaller studies report higher prevalence, which might be suggestive of small study effects rather than publication bias.

**Table 6:** Factors associated with depression in TB patients in India

Themes	Predictors reported
Gender	Female [48]
Demographic	Age group (20–39) [50]
Education	Literate [40], Educated more than high school [48], More years of education [33, 42]
Occupation	Being unemployed [28, 39, 43, 47], Working group [44]
Marital and relationship issues	Less family size [45], Disturbed marriage and interpersonal relation issues [47], Family issues, Being single [37], Unmarried [39, 41]
Religion	Hindu[40]
Socio-economic status	Lower and middle class categories [28], Financial/other troubling issues [34], Low socioeconomic status [28, 37, 49], Above Poverty Line [48], Low household income [49], Upper socio-economic group [44]
Habitation	Urban inhabitants [41, 48]
Disease related	MDR-TB cases [41], Clinical variables[30], Duration of TB infection/illness [30, 33], Pulmonary TB [30, 44, 50]
Treatment related	Longer treatment duration [27, 40], Relapsed cases [47], Greater severity of the illness [27], Type of treatment [30], Sputum status [30], Complications of pulmonary TB [30], Presence of adverse drug event [7, 34], Being on TB treatment [37], In continuation phase of treatment [48], First four months of treatment [50], Treatment non-adherence [48], Previously treated [41]
Social factors	Being homeless [46], Perceived social isolation [44], Discrimination [34], High perceived stigma [49], Low social support [49]
Behavioural	Alcoholics [50]

## Discussion

The meta-analysis estimated the prevalence of depression in TB patients in India at 37% (95% CI: 26- 49%). Previous systematic reviews and meta-analyses have reported varying degrees of prevalence of depression in different types of TB cases across various study settings around the globe. A study in South Asia estimated the prevalence of depression among DR-TB cases at 54% (95% CI: 42–65%) [23], whereas another study that has included studies from different parts of the globe estimated the pooled prevalence of depression in TB patients at 11% (95% CI: 11–12%) [22]. A meta-analysis that pooled data from 40 studies across 20 countries estimated the prevalence of depression at 25% (95% CI: 14–39) [51]. With pooled data from 25 studies across seven countries with 4903 participants, a meta-analysis estimated the prevalence of depression in TB patients at 45.19% (95% CI 38.04–52.55) [11]. In addition to meta-analysis, several observational studies have estimated the prevalence of depression in TB patients. One of the cross-sectional studies that analysed data from 48 low and middle-income countries estimated the prevalence at 23.7%. The study further reported that TB was associated with 1.98 (95% CI 1.47–2.67) times higher odds for subsyndromal depression, 1.75 (95% CI 1.26–2.42) for brief depressive episode and 3.68 (95% CI 3.01–4.50) for depressive episode [52]. Another Institution-based cross-sectional study among 403 TB patients in Ethiopia estimated the prevalence of depression in TB patients at 51.9% (95% CI = 42.7, 62.2%) [53]. Compared to the present study estimate, a clear variation is being observed in the estimation of the prevalence in TB patients, which is mainly due to the geographical settings, tools used for estimation, sample size and cultural contexts. However, what is important to note here is that the estimated prevalence of depression in TB patients in India underscores a relatively higher burden among the Indian population. This estimation is again much higher than the estimation of the World Health Organisation, which is reported at 5% (4% among men and 6% among women) [54]. 

A subgroup analysis based on the types of TB cases indicated prevalence of 39% (CI: 26-54%) in both DR and DS TB cases, followed by DR- TB cases [36% (CI: 09- 68%)], DS- TB cases [32% (CI: 14- 53%)]. This meta-analysis found slight differences in prevalence across different types of TB cases, all cases being the highest, followed by DR and DS TB cases. A recent prospective study over 10 years (2010–2020) among 224 participants in the Dobrogea region, Romania, reported major depression in MDR-TB cases compared to the DS-TB cases [55]. Two recent systematic reviews estimated a higher prevalence of depression in DS-TB cases [22] and, in general, all TB cases compared to the DR-TB cases [56]. Although this review estimated a slightly higher prevalence in all types of TB cases in general, the slightly higher prevalence of depression among DR-TB cases, compared to DS-TB cases, estimated through this review, could be attributed to the chronicity of the ailment. The DR-TB cases undergo a protracted duration of treatment with a very complex regimen. This sort of demanding treatment regimen is most likely to impose substantial psychological strain as compared to their DS-TB counterparts [57]. Furthermore, the chronicity of DR-TB leads to amplified stress and anxiety, culminating in higher rates of depression among DR-TB cases [58]. These findings emphasise how critical it is to treat both the physical and related mental health issues of DR-TB cases. To promote better overall health and treatment outcomes of DR-TB cases, comprehensive care measures should be developed to lessen the psychological effects of chronic morbidity [23]. 

The study also found that one specific group of TB patients had the highest prevalence of depression at 96%, who were homeless P-TB cases. A study in Los Angeles among 415 homeless adults with latent TB found that approximately 50% (*n* = 209) of the participants reported depressive symptoms [59]. Compared to this finding, the Indian finding is much higher and almost double, which is largely due to the cultural setting, living conditions and the support system for this specific population group. Several observational studies have reported that globally, about 25% of homeless individuals have mental disorders [60, 61]. 1.77 million Indians are homeless, making up 0.15% of the nation’s total population, according to the 2011 Census [62]. It is estimated that between 20 and 25 per cent of India’s homeless population has a serious and chronic mental disorder [63]. A homeless person has no work, no money, no function, and little support. Since they lack an identity, they can be encountered in a variety of settings. As a result, they are not listed in any national records. Their legal existence is not proven [64]. When it comes to TB, which is already a debilitating chronic infection, it adds to the plethora of problems that the homeless population encounter on a day-to-day basis, leading to further exacerbation of mental health conditions with poor treatment outcomes for TB.

The second subgroup analysis was carried out based on the instrument used for assessing depression among the TB cases. Of the nine assessment tools used to assess depression, the pooled prevalence in studies utilising PHQ-9 was highest [43% (CI: 31- 56%)]. The possible explanation for variations in reporting depression across different study instruments could be related to the use of psychometric properties and the cut-off points (criteria) used to define depression. For instance, an assessment of the psychometric comparison between PHQ-9 and HADS-D for measuring depression severity in primary care revealed that both instruments categorised the severity significantly differently [65]. In another study that assessed the psychometric properties of PHQ-9 and BDI-II, both demonstrated adequate reliability and validity; however, they labelled the severity of depression or depressive symptoms in TB patients differently [66]. Furthermore, depending on the study and the cutoff score, the PHQ-9 and HADS have different sensitivity and specificity for depression. However, when applying the cutoff ≥ 10, the PHQ-9 generally has a specificity range from 77 to 88% and a sensitivity of about 90%. The HADS has a predictive validity for identification of roughly 70% and a sensitivity and specificity of about 80% [67, 68]. 

The review identified several factors associated with depression in TB patients in India. These factors were categorised into twelve different categories: gender, demographics, education, occupation, marital and relationship issues, religion, socio-economic status, habitat, disease-related factors, treatment-related factors, and social and Behavioural factors. Several studies around the globe have identified several similar factors associated with depression in TB patients. A systematic review and meta-analysis pooling 19 articles with 4391 participants in TB patients of sub-Saharan Africa found that chronic illnesses, multi-drug resistance treatment, and being in the intensive phase of treatment were associated factors of depression in TB patients [69]. An observational study in North-west Ethiopia among 390 TB patients revealed that duration illness, chronicity of the disease and perceived stigma were significantly associated with depression in TB patients [70]. Similarly, another Ethiopian study reported that Extra-pulmonary TB, poor social support, and perceived stigma were associated factors for depression in TB patients [71]. A facility-based cross-sectional Chinese study among 219 TB patients revealed that age, difficulty in healing, fear of illness, stigma, feeling discriminated against, physical condition and previous anxiety and depression were associated with depression in TB patients [72]. Being an infectious disease, many times these factors were not considered by the treating primary care physician or the specialists [7]. This happens most commonly as the primary goal of treating a case of TB is directed towards improving physical health, ensuring drug adherence, and managing adverse drug reactions to achieve a negative laboratory report of M-TB presence in the patient’s body. However, overlooking the psychological changes and the needs of the patients can lead to negative outcomes such as treatment discontinuation or loss.^[74]^

The findings of this study provided valuable information about the prevalence of depression in TB patients in India. India, as a country, aims to eliminate TB by 2025, five years ahead of the Sustainable Development Goal targets, emphasising the political will and health system preparedness for the same. However, simply focusing on the physical health of TB patients may not be sufficient, and a strategic focus on both physical and mental health and proper arrangements for the same would go hand in hand with achieving the elimination targets of TB in the country. The study findings can help healthcare providers and decision-makers in the design and implementation of targeted interventions to address both the physical and mental health of TB patients in India.

### Implications of the study

The study has several implications, which can be broadly categorised into three. **(a) Policy implications**: The study highlights targeted mental health interventions for TB patients at all levels of the health system, preferably at the level of primary healthcare. Counselling of all TB patients should be integrated with the National TB Elimination Program. **(b) Health system Implications**: Given the dearth of mental health professionals in the health system, especially at the primary health care level, however, the scope should be created to strengthen the health systems towards this so that an integrated approach towards several chronic diseases can be instituted. At the current arrangements where the Community Health Officers are trained on comprehensive primary health care, they should be utilised to provide mental health support to TB patients at the Health and Wellness Centre level. (c) **Community implications**: When it comes to mental health, the community plays a very critical role. In the case of TB, it becomes more prominent as stigma is the strongest predictor, which can only be alleviated with the support of family and the community.

### Strengths and limitations of the study

This systematic review and meta-analysis is likely the first review on depression in TB patients in India. However, similar reviews have pooled data from Indian studies. The study used a predefined search strategy, performed data extraction, and evaluated quality by two researchers. This minimised bias in the review process. The included studies underwent quality assessment based on the Joanna Briggs Institute Critical Appraisal checklist for cross-sectional studies. The performance of subgroup analysis, Egger’s test, the trim-and-fill method, and the leave-one-out sensitivity analysis represented the major strengths of this review.

There are several limitations to the study. The meta-analysis reported high heterogeneity (I^2^-99.10%), suggesting variability in the included data. Secondly, although the PHQ-9 has been used the most, nine different instruments were employed to define depression, leading to varying reporting of prevalence across study populations.

## Conclusions

The review estimated the pooled prevalence of depression among TB patients in India at 37% (95% CI: 26- 49%). This estimation is more than seven times higher than the WHO level (5%) among the general population, emphasising the need for specific mental health interventions in TB patients. The study also found several predictors that are common across several studies across the globe. Notably, perceived lack of social support, stigma, and prolonged treatment duration are important considerations. It is well acknowledged that the combination of both depression and TB is a major public health problem which needs to be addressed at both the community and health facility levels.

## Data Availability

No datasets were generated or analysed during the current study.
